# Supplementary material for: descSPIM: an affordable and easy-to-build light-sheet microscope optimized for tissue clearing techniques
Source: Nat Commun. 2024 Jun 12;15:4941. doi: 10.1038/s41467-024-49131-1 (PMC11169475; doi:10.1038/s41467-024-49131-1)
Supplement: Supplementary file 1 — Supplementary Information [file 41467_2024_49131_MOESM1_ESM.pdf]

Supplementary Information for

descSPIM: an affordable and easy-to-build light-sheet microscope optimized for tissue clearing techniques

Kohei Otomo, Takaki Omura, Yuki Nozawa, Steven J. Edwards, Yukihiko Sato, Yuri Saito, Shigehiro Yagishita, Hitoshi Uchida, Yuki Watakabe, Kiyotada Naitou, Rin Yanai, Naruhiko Sahara, Satoshi Takagi, Ryohei Katayama, Yusuke Iwata, Toshiro Shiokawa, Yoku Hayakawa, Kensuke Otsuka, Haruko Watanabe-Takano, Yuka Haneda, Shigetomo Fukuhara, Miku Fujiwara, Takenobu Nii, Chikara Meno, Naoki Takeshita, Kenta Yashiro, Juan Marcelo Rosales Rocabado, Masaru Kaku, Tatsuya Yamada, Yumiko Oishi, Hiroyuki Koike, Yinglan Cheng, Keisuke Sekine, Jun-ichiro Koga, Kaori Sugiyama, Kenichi Kimura, Fuyuki Karube, Hyeree Kim, Ichiro Manabe, Tomomi Nemoto, Kazuki Tainaka, Akinobu Hamada, Hjalmar Brismar, and Etsuo A. Susaki*

* Email: suishess-kyu@umin.ac.jp

This PDF file includes:

Supplementary Figs. 1 to 19

Supplementary Table 1

Supplementary References

	Schematics	Main objects	Control	Components	Target to be placed	Price	Reference
**openSPIM**	SPIM *L-: conventional; X-: with dual-angle illumination and detection	Living small samples	Micromanager-based	Custom-made parts included	Laboratory	21k USD- (L-) 35k USD- (X-) single ex. wavelength and sCMOS camera excluded	Pitrone P. G., et al., Nat. Methods (2013); Girstmair J. et al., Adv. Biol. (2021).
**OpenSpin Microscopy**	SPIM, DSLM and OPT	Living small samples and meso-scaled samples	Micromanager-based	Custom-made parts included	Laboratory or Facility	NaN	Gualda E. J. et al., Nat. Methods (2013).
**mesoSPIM**	SPIM with axial sweeping and dual angle illumination and detection	Meso-scaled cleared tissues and animals	Python and PyQt5-based	Custom-made parts included	Facility	162k USD version 5 with 4-ex. wavelength	Voigt F. F., et al., Nat. Methods (2019); Valdimirov N., Voigt F. F., et al., Nat. Commun. (2024)
**Benchtop mesoSPIM**	SPIM with axial sweeping and dual angle illumination and detection	Meso-scaled cleared tissues and animals	Python and PyQt5-based	Custom-made parts included	Facility	95k USD with 3-ex. wavelength	Valdimirov N., Voigt F. F., et al., Nat. Commun. (2024)
**descSPIM**	SPIM	Meso-scaled cleared tissues	Devise-associated software	All commercially available parts	Laboratory or personal	20k-50k USD depending on the number of ex. wavelength (1-4)	This paper

Supplementary Fig. 1. Comparison of open-source light-sheet systems.

A chart comparing open-source light-sheet microscopy. descSPIM is the only instrument capable of balancing cleared tissue imaging with an affordable price. descSPIM also excluded the use of custom-made components for user convenience.

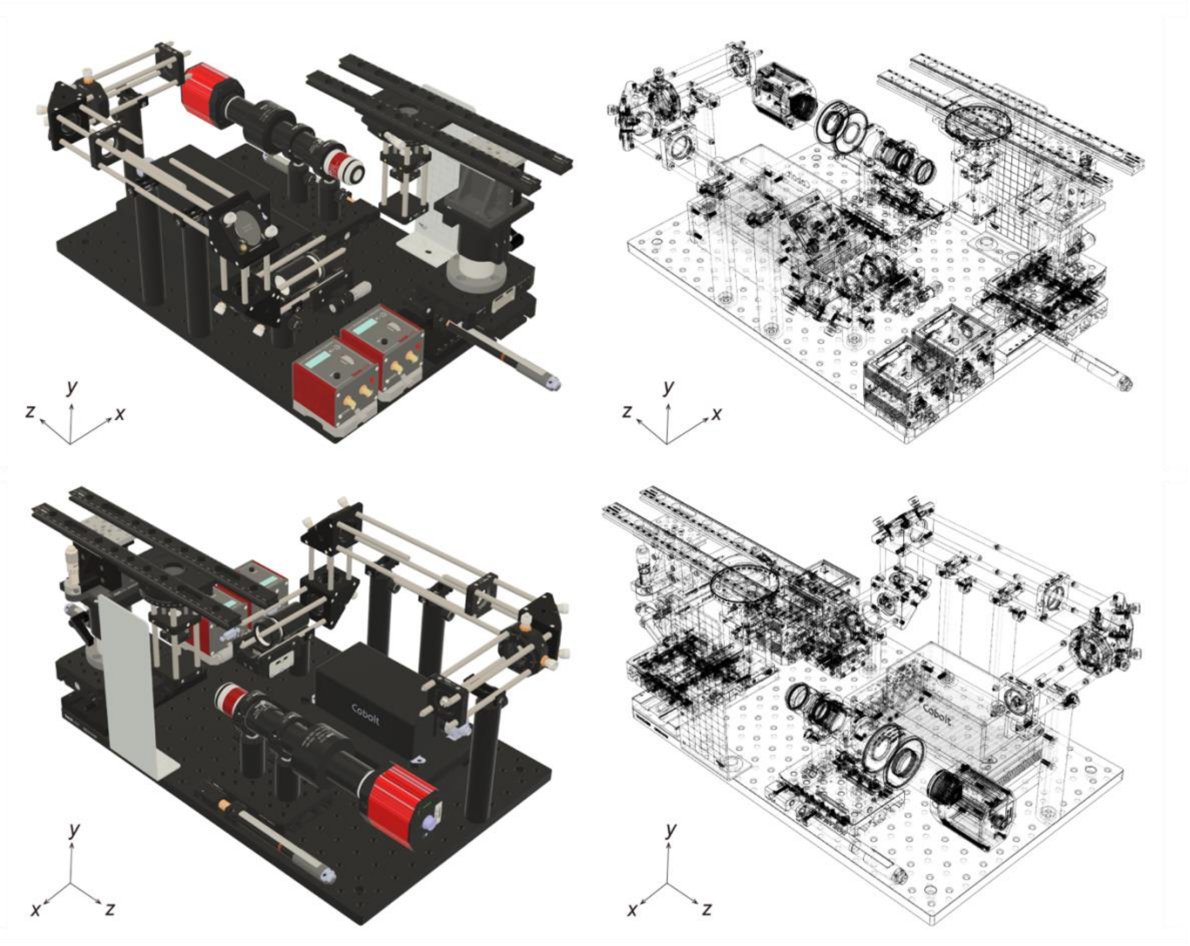

Supplementary Fig. 2. Schematic overview of the descSPIM.

Three-dimensional geometries of the descSPIM system viewed from the opposite direction, both in shaded mode (left) and wireframe mode (right).

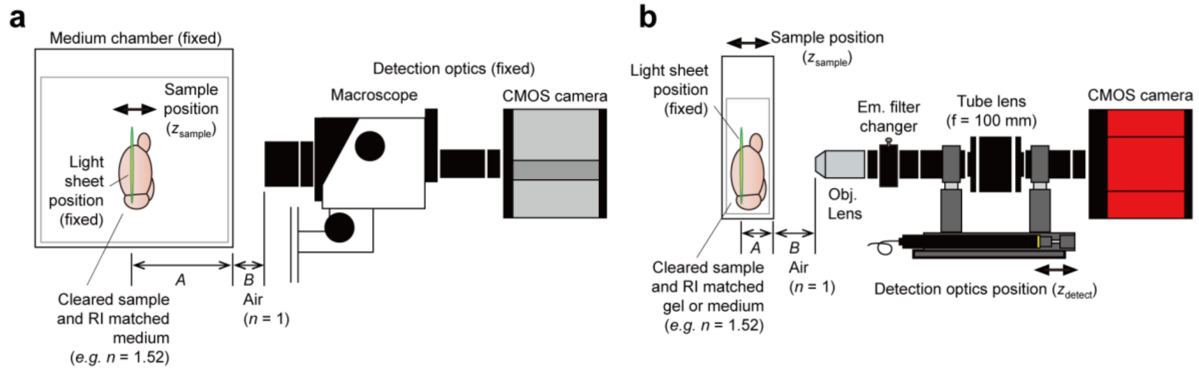

Supplementary Fig. 3. Implementation of focus tracing.

a. A conventional system with a positionally fixed medium chamber fulfilled with a clearing reagent or a RI-matched immersion oil (e.g., *n* = 1.52). The sample is moved along the *z*-axis within the chamber. In this case, the ratio of *A* (the distance from the chamber wall to the light-sheet illumination, with RI of the immersion reagent) and *B* (the distance from the objective lens to the chamber wall, with RI of the air (1.0)) is fixed.

b. descSPIM sample imaging method employing a cuvette as a sample container. In this case, due to the movement of the sample chamber (the cuvette) during imaging, the ratio of *A* and *B* is altered, resulting in defocus. descSPIM applies synchronized movement of the sample stage (z_{sample}) and the detection optics (z_{detect}) to prevent the defocus. See **Supplementary Fig. 5** and **Methods** for details on how to calculate the synchronous speed correction value (the relative velocity of two actuators).

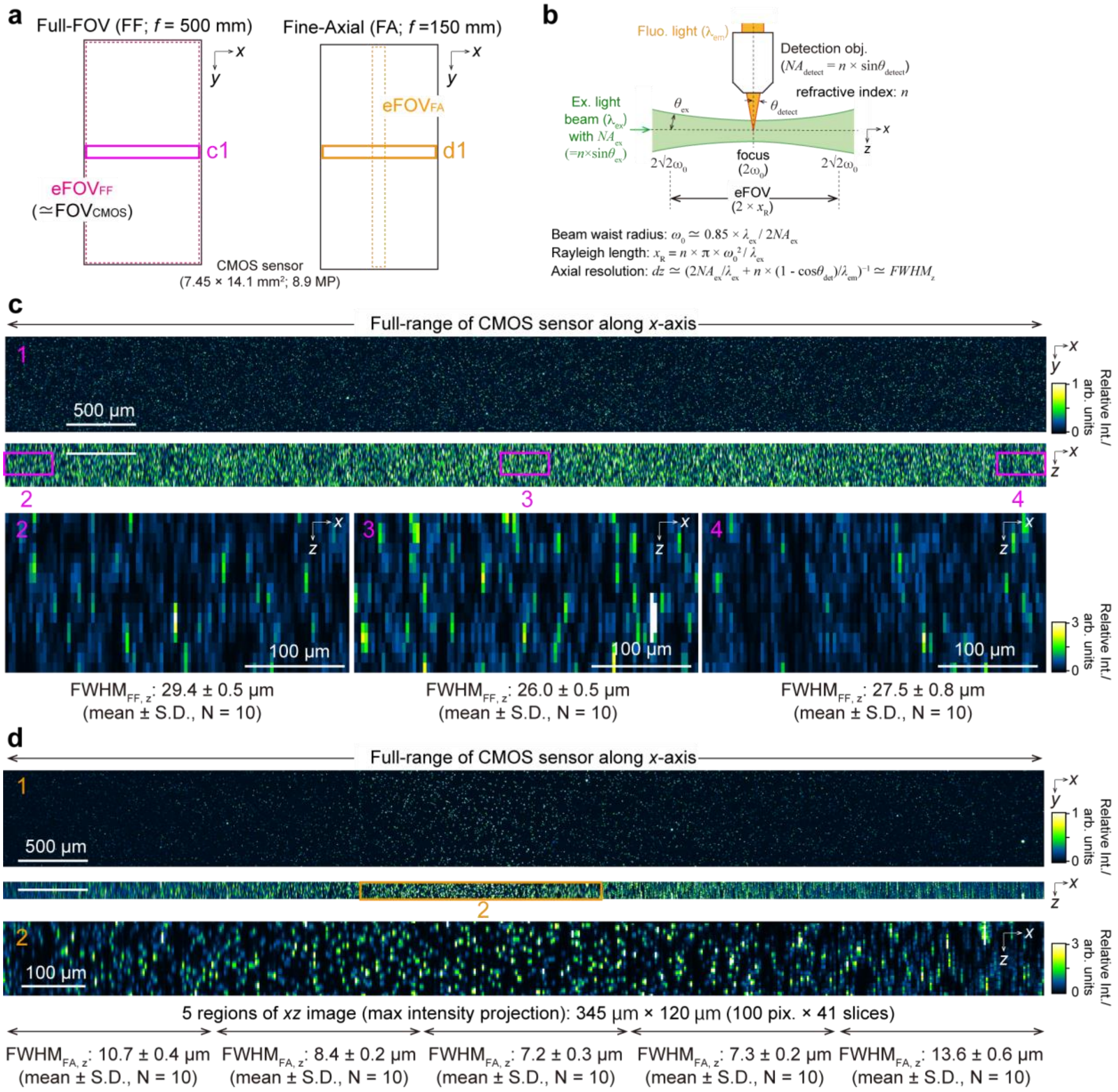

Supplementary Fig. 4. Detailed PSF calculation across the light-sheet illumination.

a. The entire CMOS sensor area and assumed light-sheet coverages by FF and FA modes are shown. **c1** and **d1** indicate the places enlarged in **c** and **d**, respectively. eFOV: effective field of view.

b. Definition of light-sheet illumination parameters from literature (1). The NA value of the cylindrical lens can be calculated with an axial resolution value (approximated as FWHM). eFOV, defined as 2 × Rayleigh length, can be then calculated with the NA value.

c. Enlarged lateral (*x*-*y*) and axial (*x*-*z*) views of the microbeads (Φ1 μm) image (max intensity projection of 300 μm in *z* range and 690 μm in *y* range) across the full-range x-axis of the CMOS sensor, captured with FF mode. Axial FWHMs measured at the center and margins indicate that the light-sheet thickness is nearly homogeneous (approximately 26–29 μm) across the range.

d. Enlarged lateral (*x*-*y*) and axial (*x*-*z*) views of the microbeads image (max intensity projection of 120 μm in *z* range and 690 μm in *y* range) captured with FA mode. At the sheet-focused position (the center of box 2), the axial FWHM is approximately 7.2 μm. The eFOV can be regarded up to the area where the FWHM is under ~10.2 μm ($\sqrt{2} \times 7.2$ μm, based on the definition of Rayleigh length). The image indicates that the FWHM ranges between 7.2–10.2 μm over a width of 300 pixels. An estimate of the eFOV from the effective NA value is 830 μm, which is consistent with the result in panel 2.

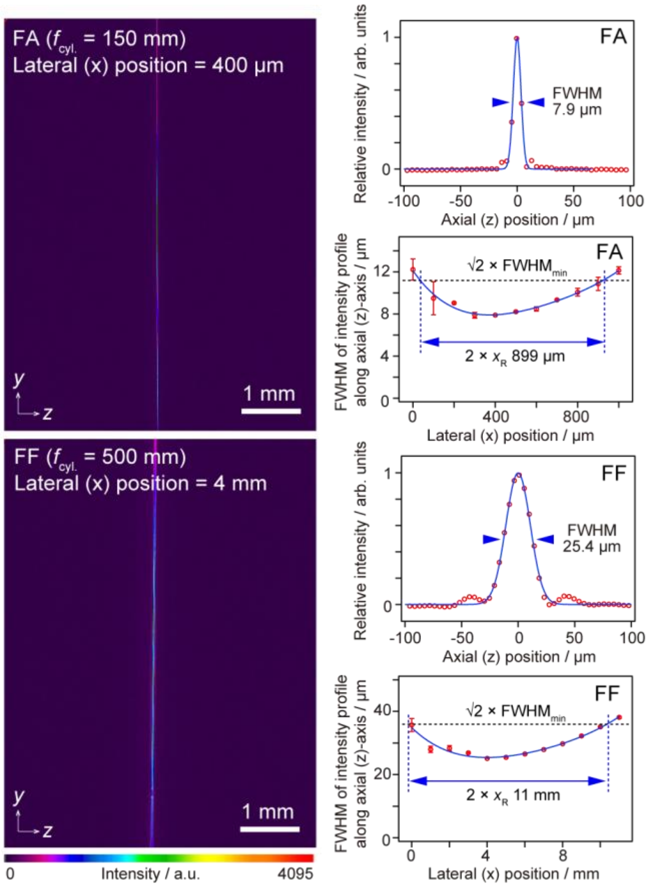

Supplementary Fig. 5. Light-sheet shapes directly measured by a beam profiler. Left panels show beam profiles near focal positions of excitation light-sheets in FF and FA modes. Right panels show axial intensity profiles at the peak position (assuming the beam profile of the thinnest position of the light sheet) and the FWHM values along lateral (*x*) axis (assuming the light-sheet shape as shown in **Supplementary Fig. 4b**) in upper and lower panels, respectively. The FWHM values along the lateral axis were fitted by 5th polynomial functions, considering their asymmetric dependencies to the lateral position derived from spherical aberrations. Measured values of beam waist sizes and 2× Rayleigh lengths are consistent with the values estimated from PSFs in **Supplementary Fig. 4.**

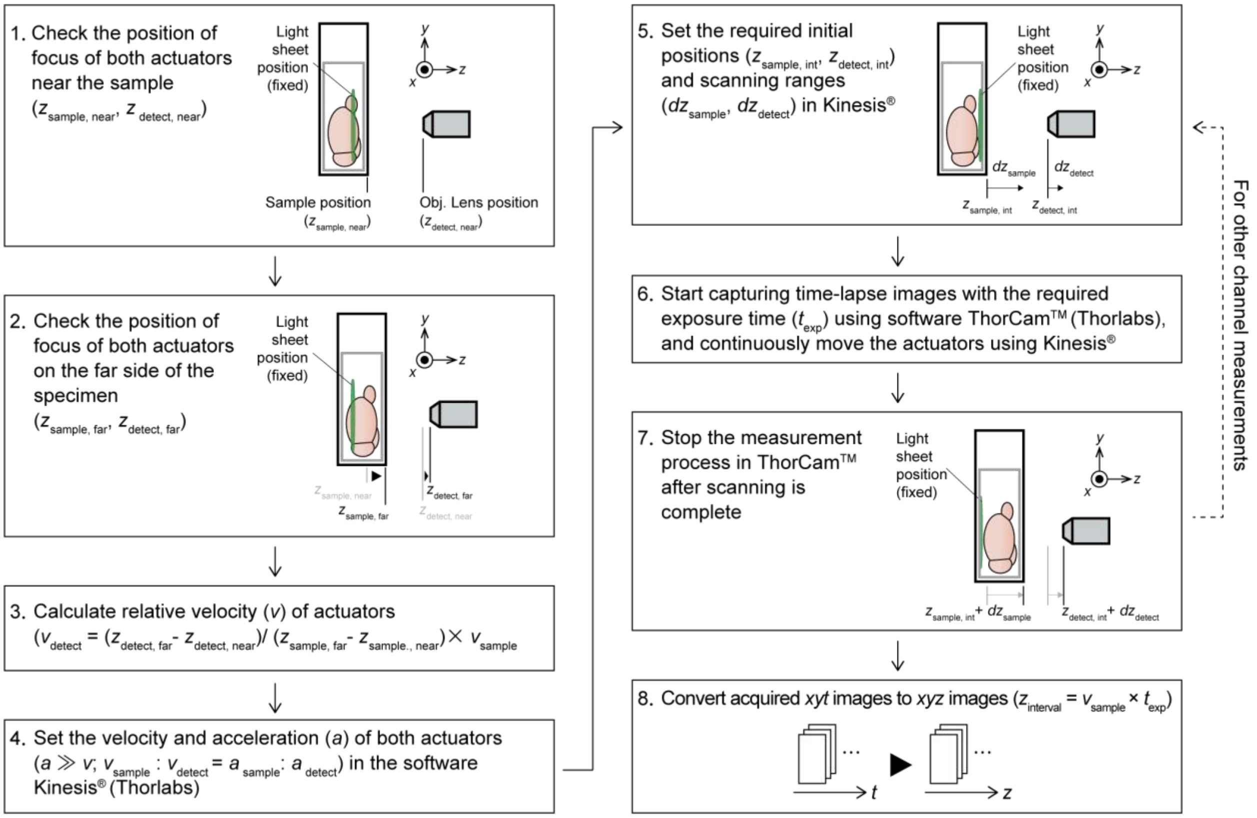

Supplementary Fig. 6. Operation workflow.

The steps 1–3 are for calculating the synchronous speed correction value (relative velocity of actuators). Although this value should be theoretically estimated as in **Supplementary Fig. 3**, it can be influenced by the actuators' capacity for weight and the PC's specifications (see **Methods**). For calculating the value in the practical situation, an operator moves the sample stage to the z-stack start position and gets the stage position. Then, the operator moves the detection objective to the focused position, and also gets the actuator position (step 1). The operator repeats the same steps at the *z*-stack end position (step 2) and calculates the relative velocity of the actuators (step 3). The velocities, accelerations, initial positions of these two actuators, and their scanning ranges are set on the associated software (Kinesis®, Thorlabs) (steps 4, 5). After the laser illumination is turned on, the operator starts image acquisition by clicking the start buttons of the Kinesis® and the camera-associated software (ThorCam™, Thorlabs). The *z*-stack is collected as a time-lapse (*xy*-*t*) file (steps 6, 7). Finally, the acquired movie is converted

to a z-stack (xy-z) file with ImageJ/Fiji. The z-interval is calculated as the moving speed of the sample stage actuator (v_{sample}) × exposure time (t_{exp}) (step 8).

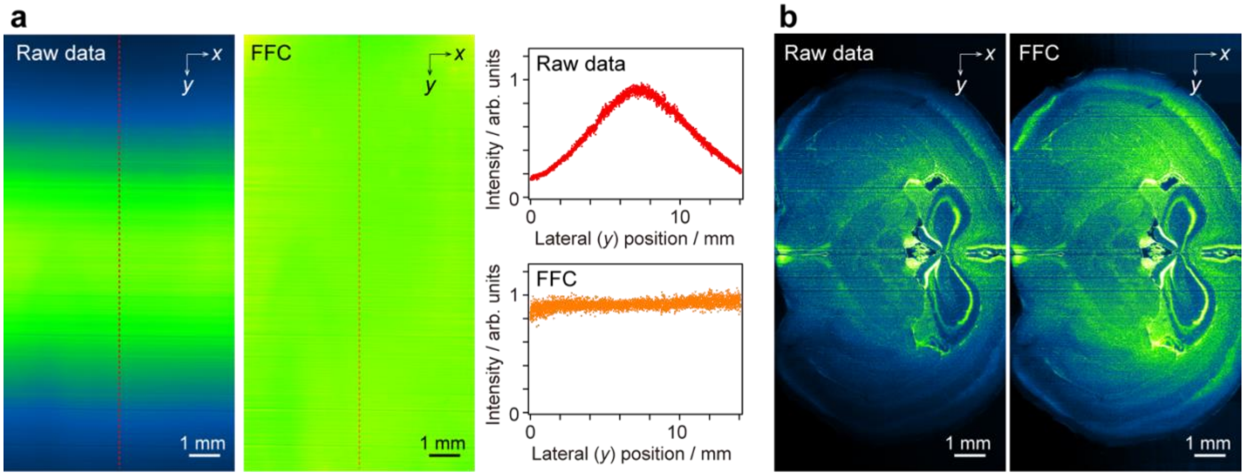

Supplementary Fig. 7. Flat-field correction (FFC) of Gaussian illumination.

a. FFC implementation (2). A fluorescein-dissolved clearing reagent was imaged as a reference. The Gaussian-shaped intensity profile was converted into a flat intensity profile by our custom ImageJ macro code (see **Methods** and our GitHub website: https://github.com/dbsb-juntendo/descSPIM).

b. An FFC example applied to the actual sample image. A coronal image of a 2 mm-thick mouse brain section stained with PI was obtained, and the FFC was applied.

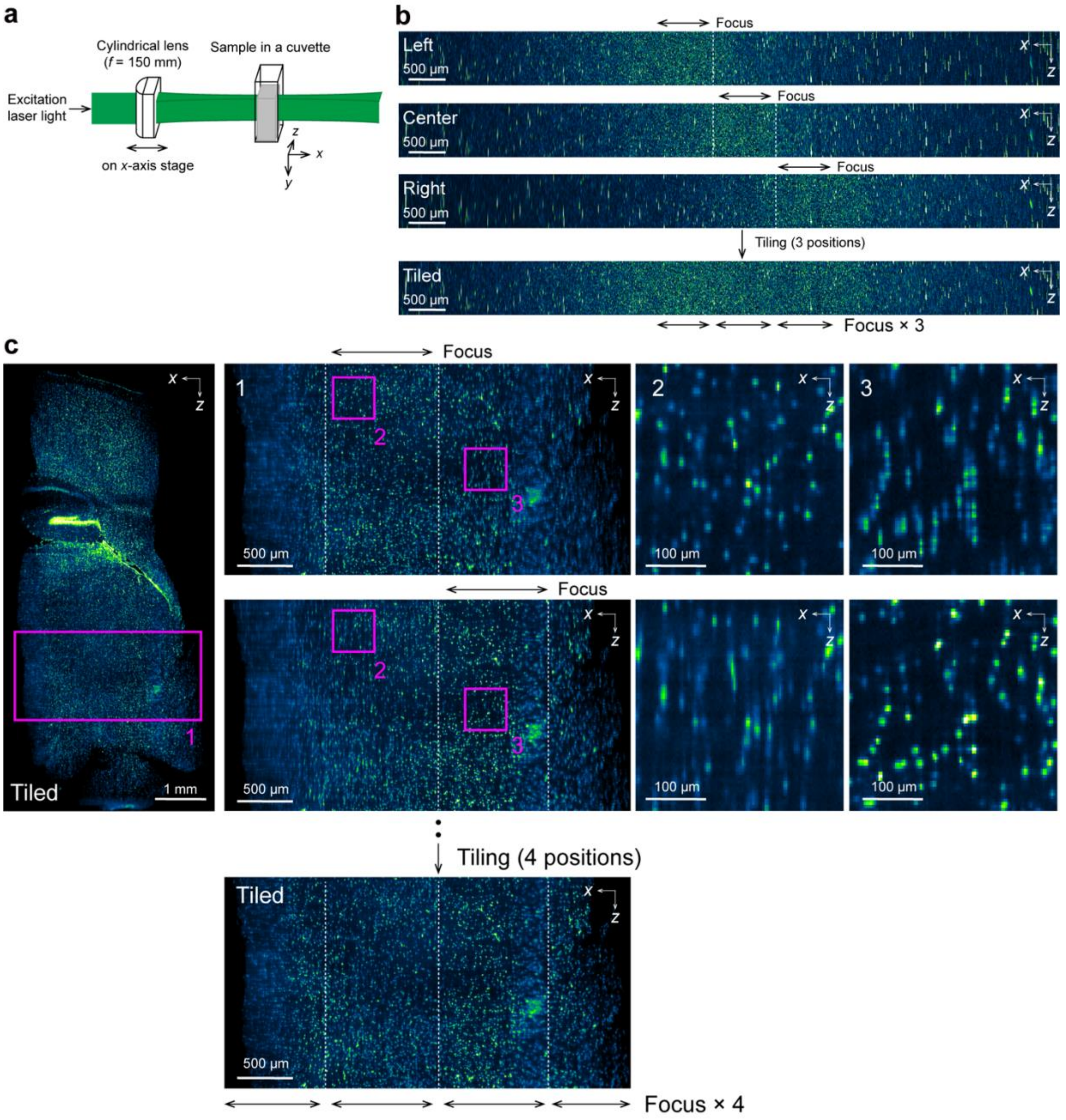

Supplementary Fig. 8. Adaptation of tiling light-sheet method.

a. The imaging scheme. descSPIM adopts the tiling light-sheet method (3) by manually moving the cylindrical lens (*f* = 150 mm) for FA illumination mode.

b. TLS procedure. The axial image of the bead signals (max intensity projection of 690 µm in *y* range) showed a high signal contrast area corresponding to the eFOV (practical 2× Rayleigh length) of the sheet illumination. Based on the estimation in **Supplementary Fig. 4**, we moved the cylindrical lens from the left (proximal side of the lens) to the right

(distal side of the lens) by 500 µm in the air (corresponding to 760 µm in the medium with RI = 1.52) in each step. The tiling position was identified based on the signal contrast calculation with the Fourier function of ImageJ (see **Methods**).

c. An example of TLS adoption for PI-stained 2 mm-thick mouse brain data.

The dotted lines in **b** and **c** show the tiling positions on the *x* axis.

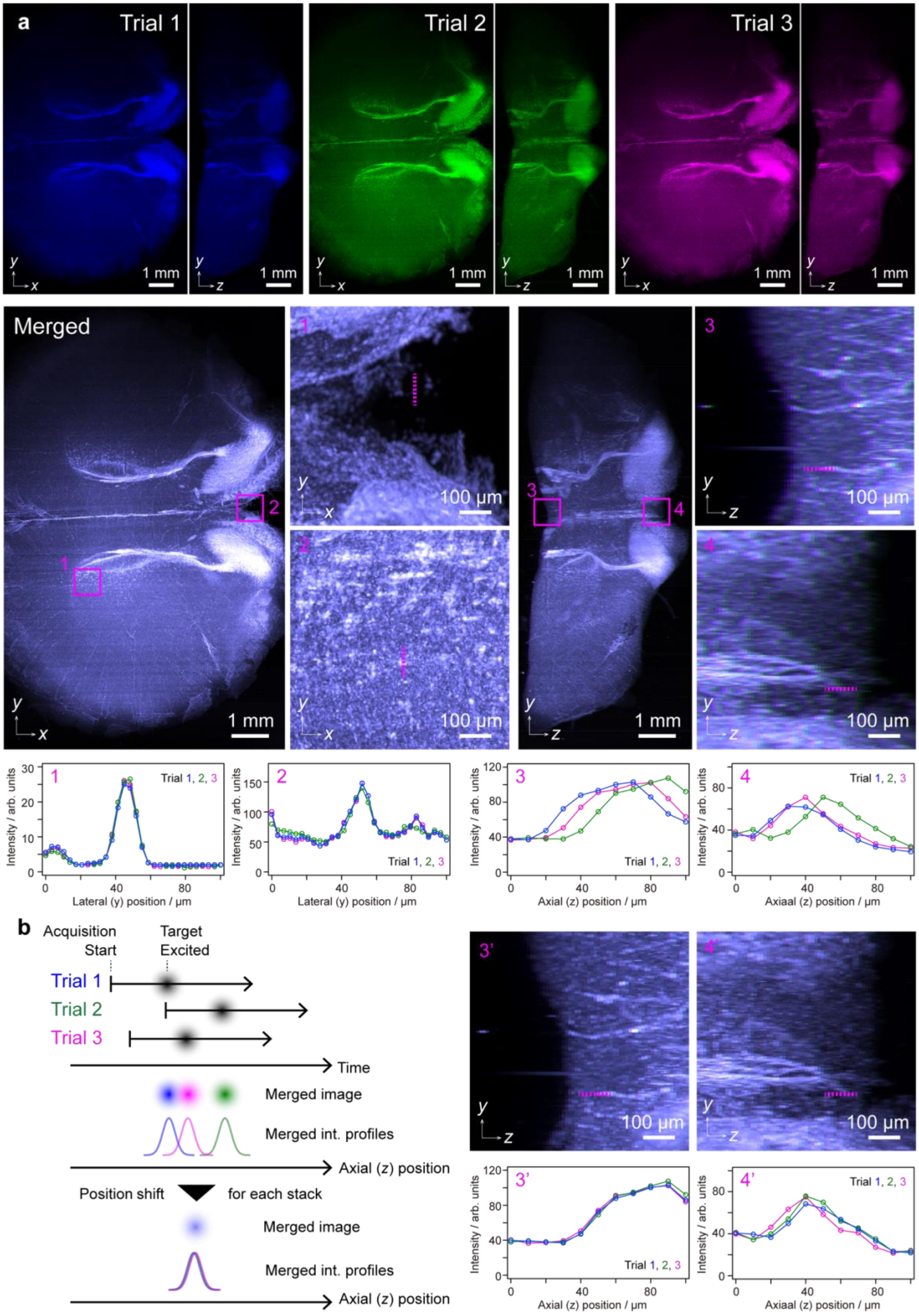
a
Trial 1
Trial 2
Trial 3
1 mm
Merged
100 µm
Lateral (y) position / µm
Axial (z) position / µm
Axiaal (z) position / µm
Intensity / arb. units
Trial 1, 2, 3
b
Acquisition Start
Target Excited
Trial 1
Trial 2
Trial 3
Time
Merged image
Merged int. profiles
Axial (z) position
Position shift
for each stack
Merged image
Merged int. profiles
Axial (z) position

Supplementary Fig. 9. Reproducibility of *z*-stack measurements using two device-associated software.

a. Three independent *z*-stack acquisitions were performed using ThorCAM™ and Kinesis® on a 2-mm thick mouse brain slice stained with PI (**Supplementary Fig. 6**), and the images were merged. No lateral shifts were observed in all three trials, while axial shifts of approximately 1 to 2 slices occurred. Axial shifts were largely maintained near the surface at the beginning of imaging and near the back at the end of imaging.

b. By shifting the slice position by 1 or 2 slices in each of the three *z*-stacks, we successfully corrected the shifts in all three trials from the surface to the back.

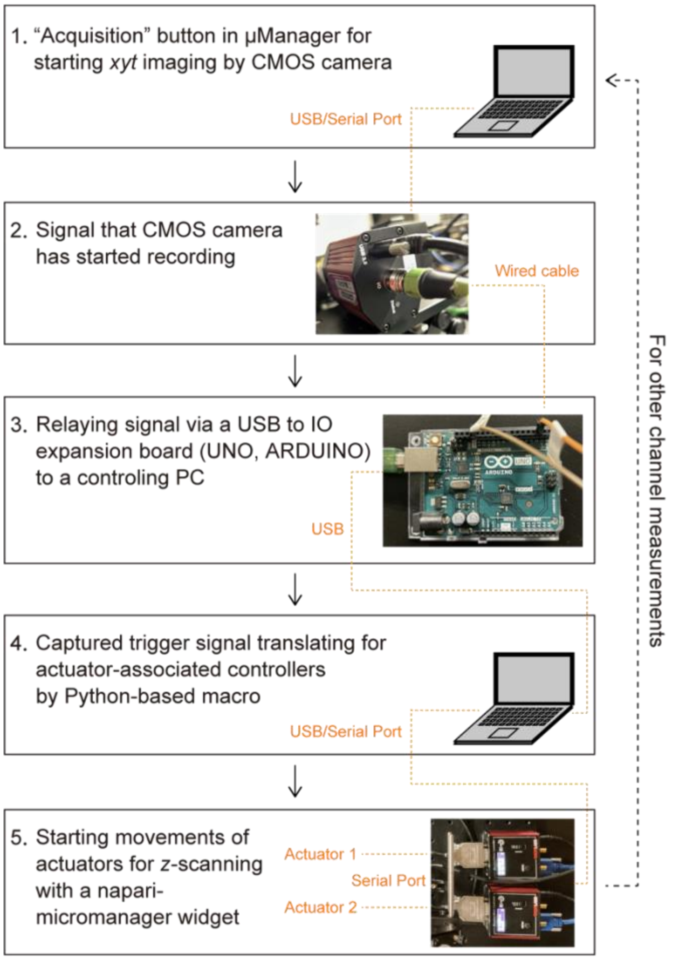

Supplementary Fig. 10. Advanced operation workflow

Based on general operational workflow (**Supplementary Fig. 6**), a low-cost "USB to IO" expansion board was used to synchronize the initiation of camera acquisition with stage movement to accomplish *z*-stack acquisition automatically. In Step 1, µManager starts the *xyt* image acquisition of the CMOS camera. The signal from the camera "REC started" is sensed by the board (Step 2), which converts it into a trigger signal (Step 3). The trigger signal activates two actuator controllers via a Python program (Step 4). Concurrently manupilating actuators, precise synchronous *z*-scan is made (Step 5).

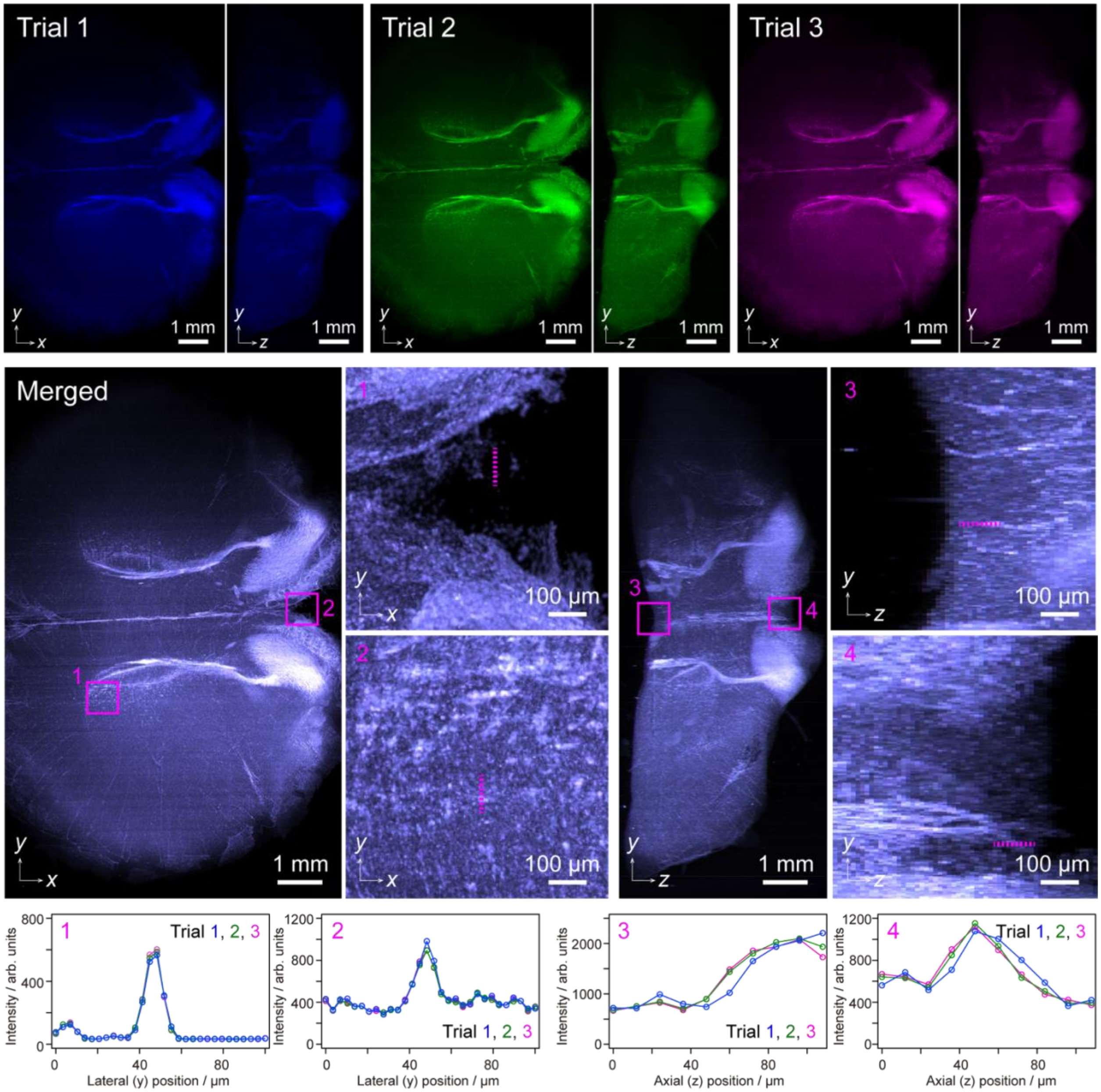

Supplementary Fig. 11. Reproducibility of *z*-stack measurements using custom-made software.

Three independent *z*-stack acquisitions were performed using µ-manager-based custom software on a 2-mm thick mouse brain slice stained with PI (**Supplementary Fig. 10**), and the images were merged. No lateral and axial shifts were observed in all three trials.

.

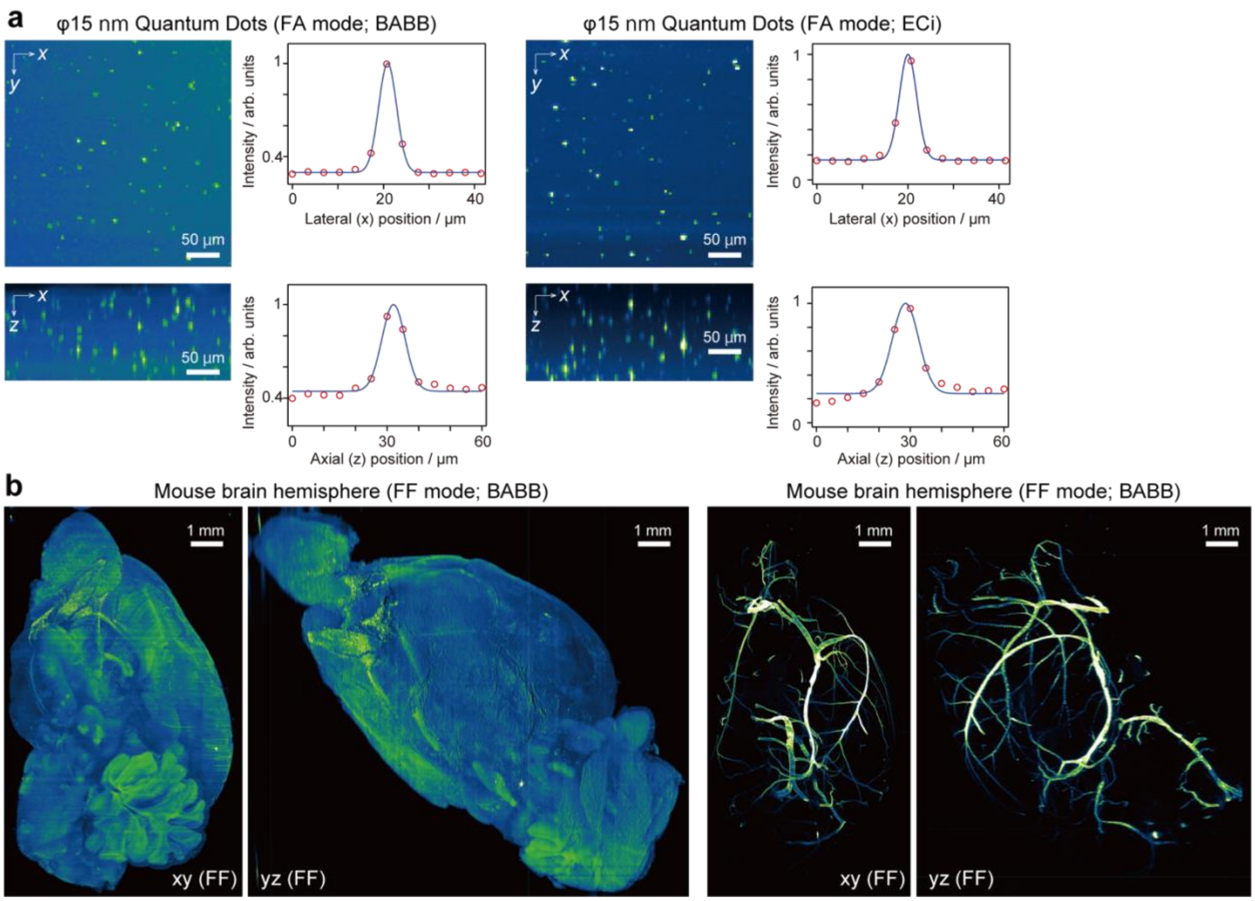

Supplementary Fig. 12. Adaptation to organic solvent-based clearing method.

a. Estimation of lateral and axial resolutions by PSFs in FA mode (evaluated with images ϕ15 nm quantum dots images). While comparable *xy* resolution was achieved in both BABB and ECi, FA mode provides approximately 10 µm of axial FWHM in both, over 30% inferior than in CUBIC-R-agarose.

b. BABB-cleared mouse hemispheres labeled with TO-PRO^{TM}-3 (cell nuclei; left) or Evans blue (blood vessels; right) were 3D-imaged with descSPIM. Full-FOV resolution was adopted. Voxel size: 3.45 × 3.45 × 20 µm^3.

The original grayscale 8-bit maps were pseudo-colored with a Green Fire Blue look-up table. Each imaging experiment for cleared biological samples was performed at least once.

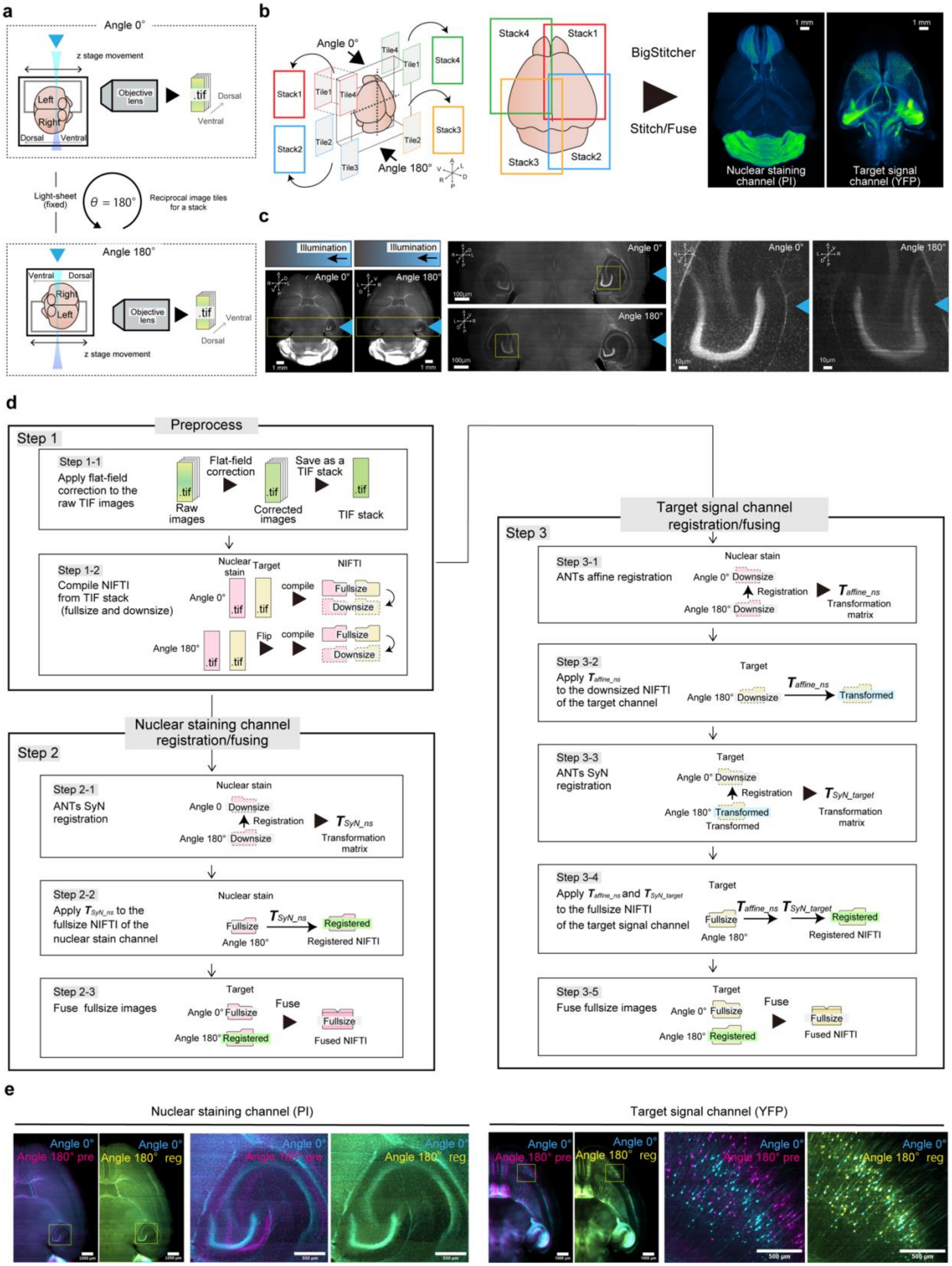
a
Angle 0°
z stage movement
Left
Right
Dorsal
Ventral
Objective lens
.tif
Light-sheet (fixed)
θ = 180°
Reciprocal image tiles for a stack
Angle 180°
b
Angle 0°
Angle 180°
Stack1
Stack2
Stack3
Stack4
Tile1
Tile2
Tile3
Tile4
BigStitcher
Stitch/Fuse
Nuclear staining channel (PI)
Target signal channel (YFP)
1 mm
c
Illumination
Angle 0°
Angle 180°
100µm
10µm
d
Preprocess
Step 1
Step 1-1
Apply flat-field correction to the raw TIF images
Flat-field correction
Save as a TIF stack
Raw images
Corrected images
TIF stack
Step 1-2
Compile NIFTI from TIF stack (fullsize and downsize)
Nuclear stain
Target
NIFTI
compile
Flip
Fullsize
Downsize
Nuclear staining channel registration/fusing
Step 2
Step 2-1
ANTs SyN registration
Registration
T_SyN_ns
Transformation matrix
Step 2-2
Apply T_SyN_ns to the fullsize NIFTI of the nuclear stain channel
Registered
Registered NIFTI
Step 2-3
Fuse fullsize images
Fuse
Fused NIFTI
Target signal channel registration/fusing
Step 3
Step 3-1
ANTs affine registration
T_affine_ns
Step 3-2
Apply T_affine_ns to the downsized NIFTI of the target channel
Transformed
Step 3-3
ANTs SyN registration
T_SyN_target
Step 3-4
Apply T_affine_ns and T_SyN_target to the fullsize NIFTI of the target signal channel
Step 3-5
Fuse fullsize images
e
Nuclear staining channel (PI)
Target signal channel (YFP)
Angle 0°
Angle 180° pre
Angle 180° reg
500 µm

Supplementary Fig. 13. Stitching and fusion of the *Thy1*-YFP-H Tg whole brain image dataset.

a. Multi-directional stack imaging (viewed from above the sample stage). The cyan triangles denote the light sheet's illumination direction. The sample stage is equipped with a θ stage to obtain multi-directional images of the corresponding sample region (0° and 180° of the left hemisphere, in this case). Registration and fusion of the stacks from the two orientations can reciprocally complement the signal intensities.

b. (Left) Reconstruction of the multi-positioned, multi-directional stack data into a single image of the whole brain. The PI nuclear staining channel images are shown. To capture the entire brain area, four image tiles were acquired at both 0° and 180° angles, respectively, for a total of eight. For instance, 'Tile 1' at an angle of 0° and 'Tile 4' at an angle of 180° corresponded spatially. The stacks for each region were then combined using the registration procedure described in **c**. (Middle) The four stacks were fused and stitched using BigStitcher (ImageJ/FIJI plugin) to generate a whole brain image for each channel. (Right) The final fused and stitched images for each color channel are shown. PI and YFP signals were detected as the nuclear staining and target signal channels, respectively. The images of the entire brain have been duplicated from **Fig. 3a**. All processes have been executed by a custom Python script, ANTs, and the ImageJ/FIJI BigStitcher plugin. Scale bars: 1 mm.

c. The stitching results of multi-positioned stacks of the PI channel acquired from angles of 0° or 180°, respectively. Cyan triangles indicate the direction of the light sheet illumination. As shown in the enlarged images in the middle and right panels, the image integrity degrades at the distant position from the illumination side. The yellow frames designate the enlarged places in the middle and right panels, respectively. Scale bars: 1 mm (left), 100 µm (middle), 10 µm (right).

d. Workflow for obtaining a registered and fused stack from the corresponding stacks from two orientations. After flat-field correction, the acquired TIF image stacks were compiled into NIfTI files (full-sized and 50%-downsized data) (steps 1-1 and 1-2). Using the nuclear staining channel as structural information, a transform matrix was then determined for each stack pair (the image downsizing was required for calculating the transformation matrices with our PC resources, see below). In order to do this, the SyN

registration of ANTs software was applied to the downsized NIfTI nuclear staining channel files (step 2-1). The resulting registration matrix (T_{SyN_ns}) was applied to the full-sized files for the transformation of the 180° stack to the 0° stack (step 2-2). The NIfTI-registered files were eventually fused by calculating the mean value of each voxel (steps 2-3). The registration and fusing of the target signal channel (YFP channel) followed a similar, though more complex, process. The first step involves applying the affine registration of the ANTs software to the downsized NIfTI files of the nuclear staining channel for brain-wide alignment (step 3-1). Then, the acquired affine matrix (T_{affine_ns}) was applied to the downsized NIfTI files of the target channel stacks (step 3-2). To precisely match the pixel-order signals in the corresponding 0° and 180° stacks, the SyN registration of ANTs software was further applied to the downsized and affine-transformed NIfTI files of the target signal channel (step 3-3) in order to acquire the second matrix (T_{SyN_target}). Both transformation matrices were then sequentially applied to the full-sized NIfTI files of the target signal channel, followed by fusion of the respective stacks (steps 3-4 and 3-5).
e. Comparison between the pre-registered (pre) and post-registered (reg) images. The yellow frames indicate the places of enlarged views. Scale bars: 1 mm (overviews), 500 µm (enlarged views).

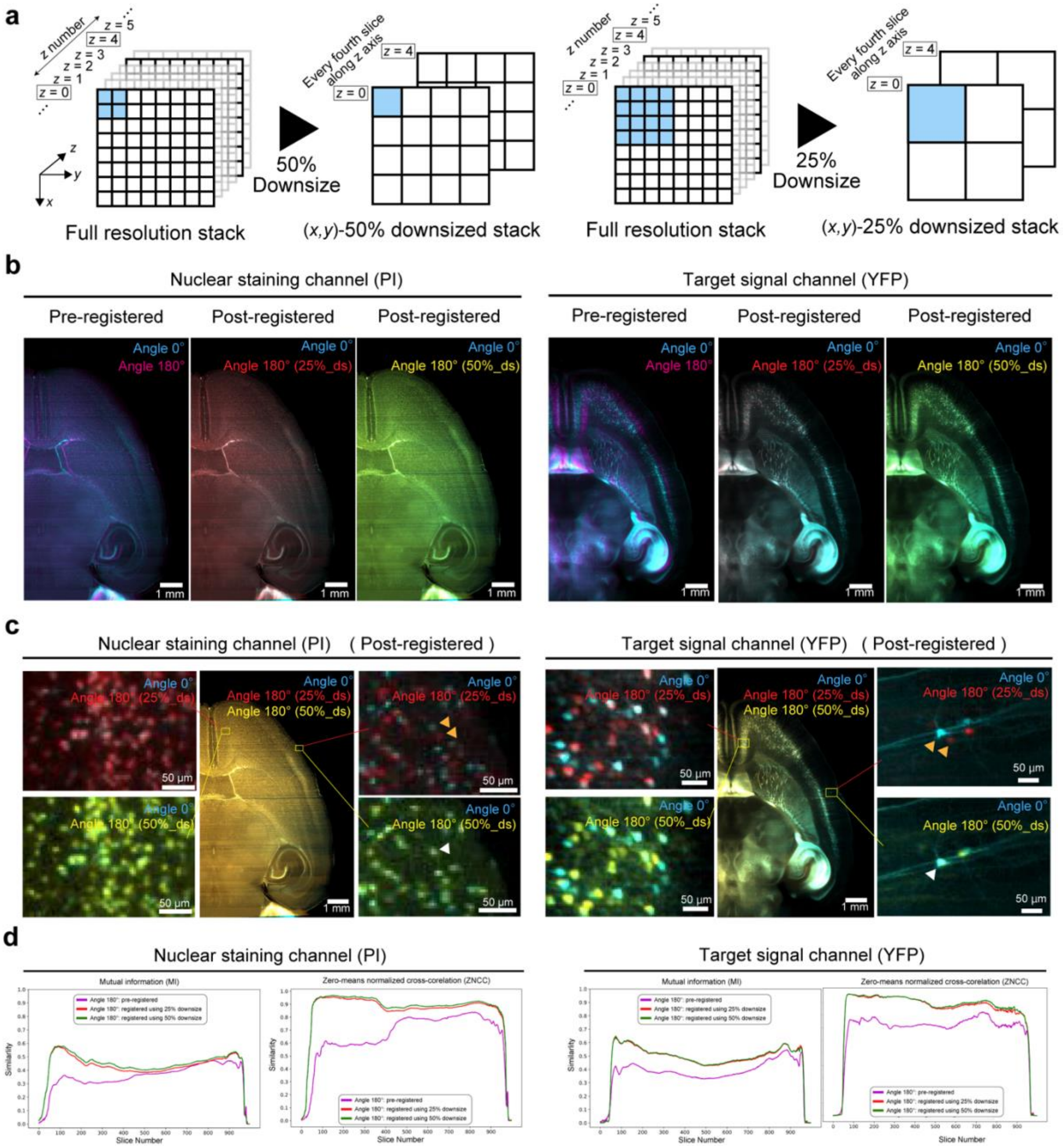

Supplementary Fig. 14. Image reduction degree and registration accuracy.

a. Reduction of image size for calculating registration matrices. The obtained images were scaled down to 50% or 25% for the *x* and *y* axes (designated 50% or 25% downsizing, respectively), resulting in four or sixteen pixels of the original image being depicted by a single pixel. Regardless of the degree of reduction, one slice out of every four was retained for the *z* axis.

b. Comparison of representative merged images of pre- or post-registered 0° and 180° stacks. The misalignment is readily apparent without registration. Regardless of the degree of image reduction, the signals from the two stacks were brain-wide aligned after registration. The nuclear staining and target signal channels display PI and YFP signals, respectively. Scale bar: 1 mm.

c. Comparison of pixel-order registration accuracy between 25% and 50% downsized data. Duplicated nuclei or neural soma observed in 25% data (orange triangles) indicate lower accuracy, while 50% downsized data (white triangles) show enhanced precision. Calculation of registration matrices for 50% downsized data required a high-end workstation (1024 GB RAM, dual 28 cores/56 threads CPUs). A decent laptop PC (64 GB of RAM, 8 cores/20 threads CPU) managed 25% data calculation. However, the PC specification was not sufficient to complete the transformation processing of the original full-sized image (~10 GB) due to memory allocation errors.

d. Quantitative evaluation of registration accuracy. Mutual Information (MI) and Normalized Zero-Means Cross-Correlation (ZNCC) were used as evaluation metrics. The accuracy of registration for both PI and YFP data was improved by using 50% downsized data compared with 25% downsized data.

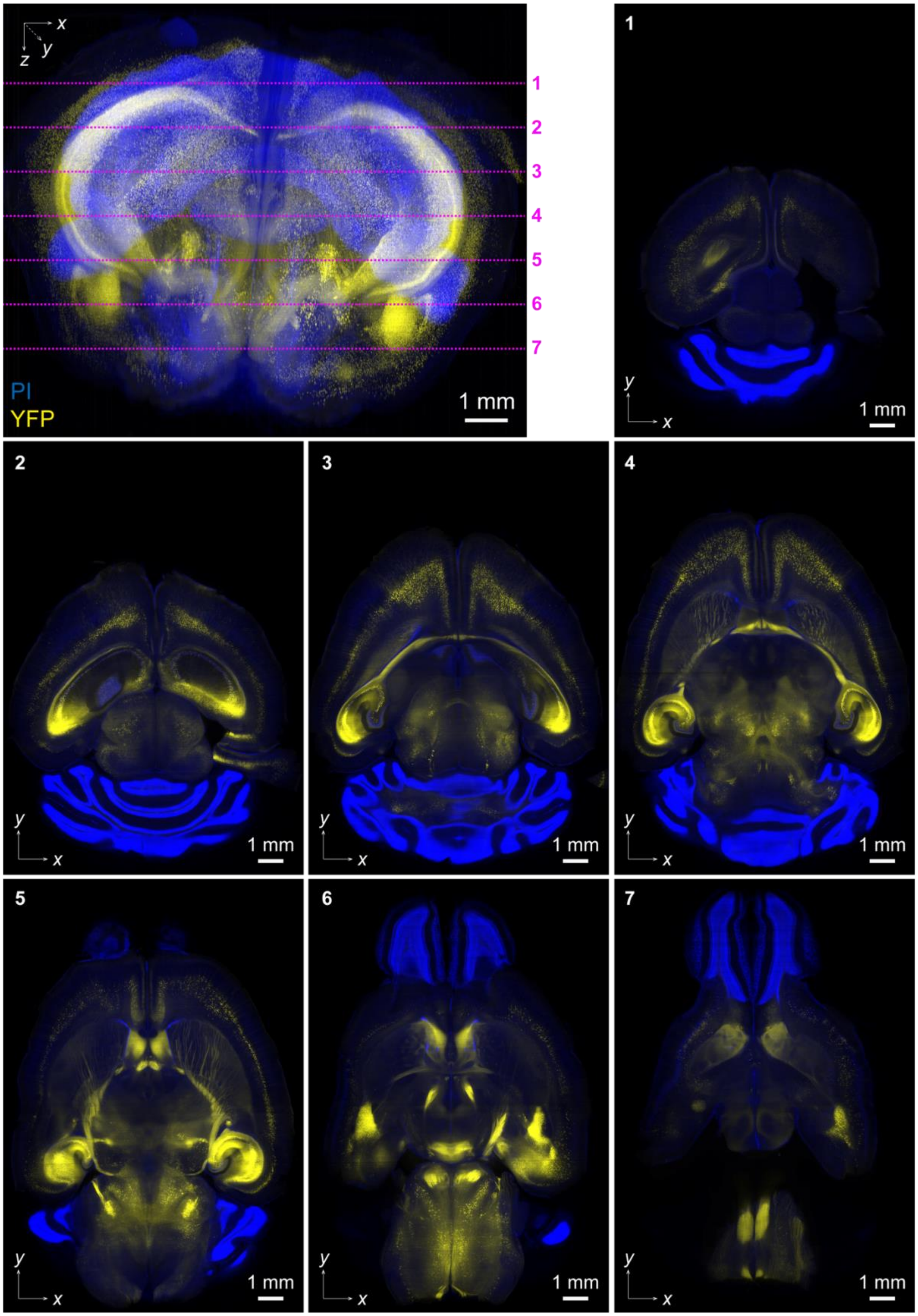
x
y
z
1
2
3
4
5
6
7
PI
YFP
1 mm
1
y
x
1 mm
2
y
x
1 mm
3
y
x
1 mm
4
y
x
1 mm
5
y
x
1 mm
6
y
x
1 mm
7
y
x
1 mm

Supplementary Fig. 15. Practical use of descSPIM in neuroscience.

A series of *xy*-plane images covering the entire PI-stained Thy1-YFP-H mouse brain using FF mode and FFC adaptation. Each slice image is a MIP with a thickness of 100 µm. Scale bar: 1 mm. Voxel size: 3.45 × 3.45 × 10 μm^3.

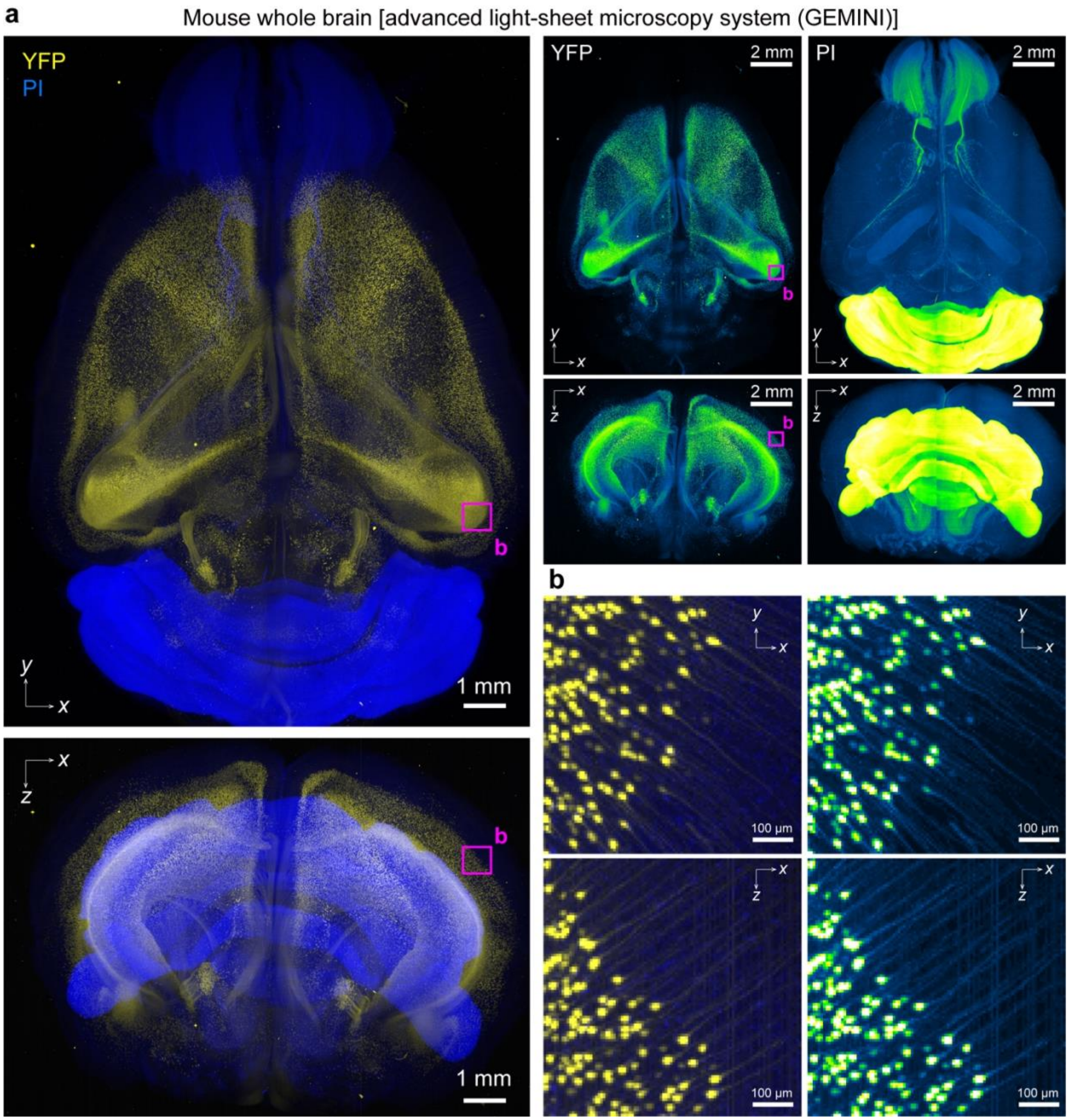

Supplementary Fig. 16. Comparative whole *Thy1*-YFP-H brain image obtained with an advanced system.

a. Whole *Thy1*-YFP-H mouse brain stained with PI was obtained with our advanced custom-build LSFM (GEMINI system) (4) as a reference for the whole-brain image obtained with descSPIM (**Fig. 3**). Voxel size: 6.5 × 6.5 × 6.5 µm^3.

b. Magnified images of YFP-expressing neurons and PI-stained nuclei within the dataset.

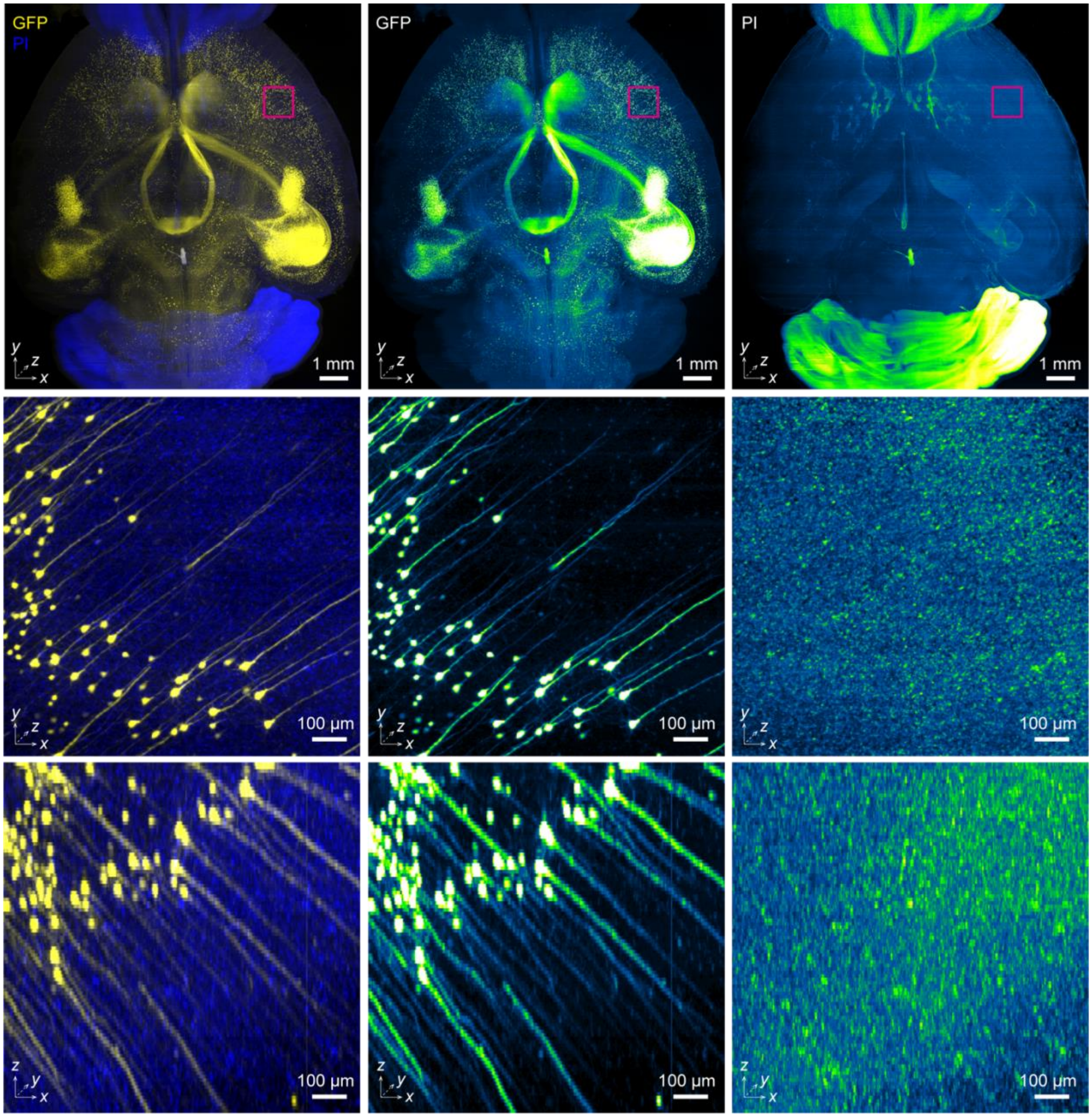

Supplementary Fig. 17. Multi-color imaging of the whole *Thy1*-GFP-M mouse brain with a single excitation wavelength.

A 3D image of the PI-stained whole *Thy1*-GFP-M mouse brain was obtained from the ventral side with FF mode and FFC adoption. Both PI and EGFP were excited with a 488 nm laser light to demonstrate a possible system case equipped with a single excitation wavelength. Four image-stack tiles were collected from a single direction. All the image tiles were stitched with BigStither. Panels show the distinct color channels obtained by a

single excitation wavelength. Voxel size: 3.45 × 3.45 × 10 μm^3. The original grayscale 8-bit maps were pseudo-colored with a Blue, Yellow or Green Fire Blue look-up table. The imaging experiments was performed twice with nearly identical results.

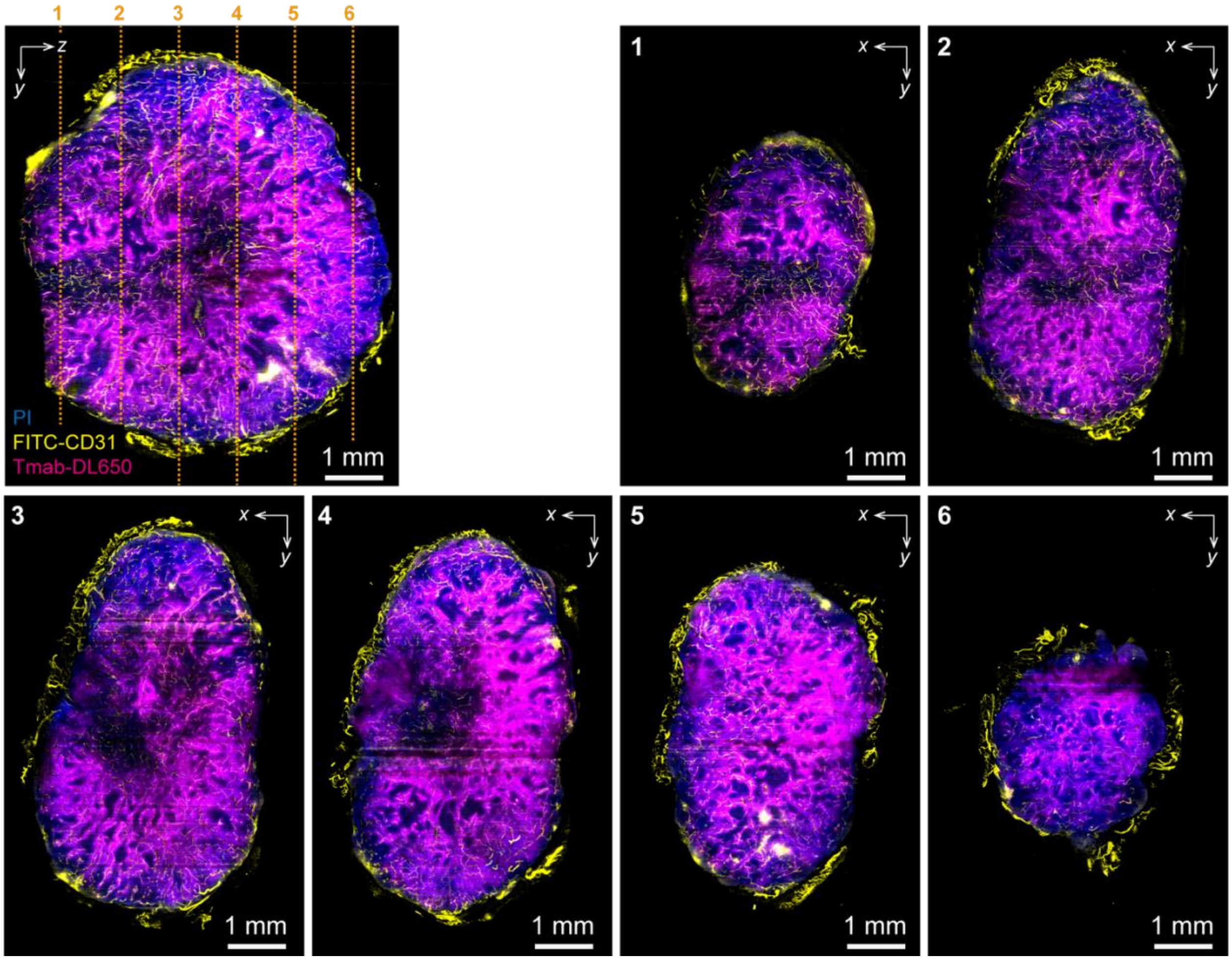

Supplementary Fig. 18. Practical use of descSPIM in the drug discovery.

A series of *x*-*y* plane images throughout the Trastuzumab-administered whole CDX mass. Vascular networks surrounding the tumor mass are obvious, while the vessels inside the tumor mass are sparser than the edge. Trastuzumab distribution varies region by region; the drug was delivered mainly in the center regions, while the peripheral areas exhibited ineffective drug distribution. Each slice image is a MIP with a thickness of 100 µm. Scale bar: 1 mm. Voxel size: $3.45 \times 3.45 \times 10\ \mu m^3$.

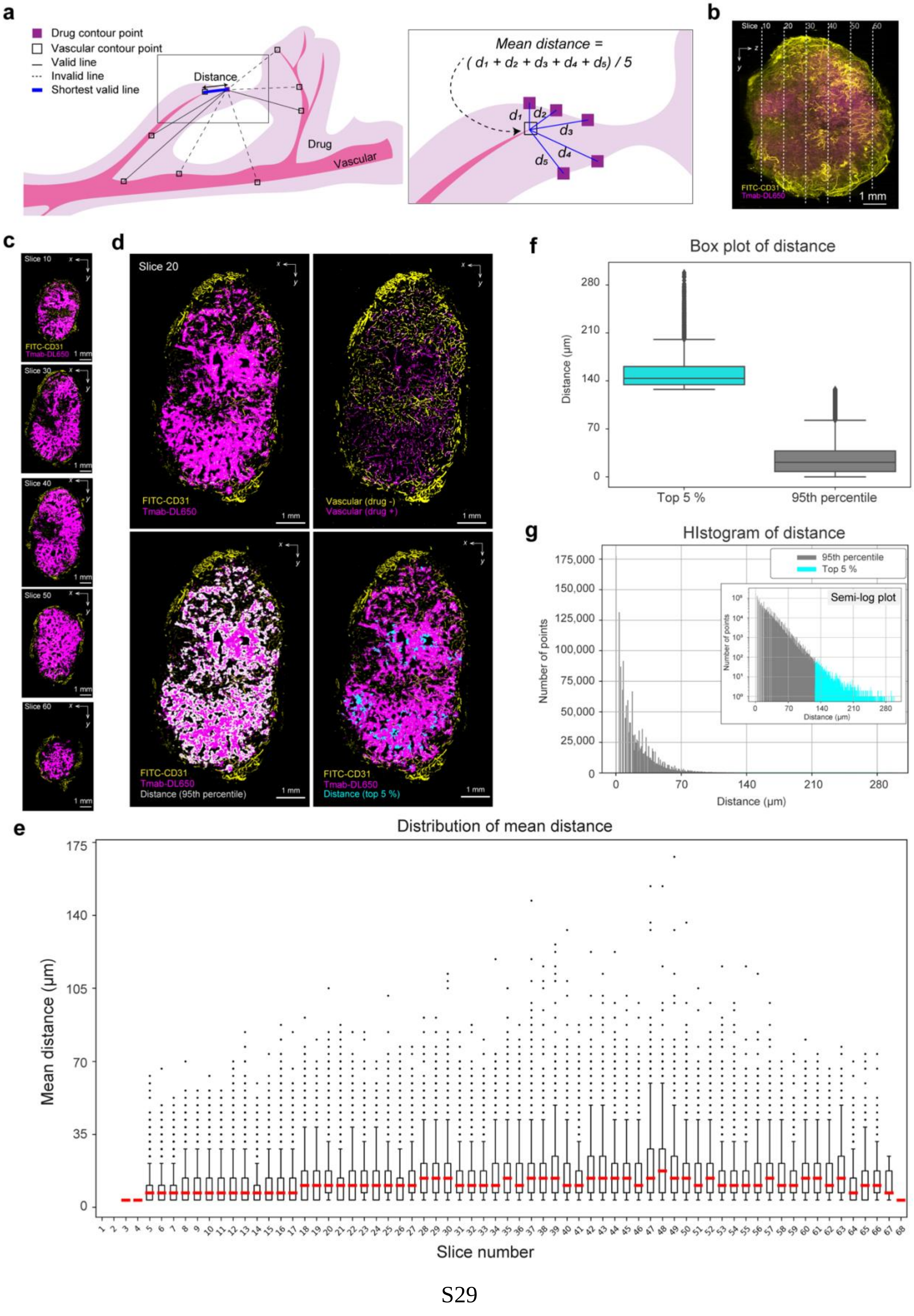
a
Drug contour point
Vascular contour point
Valid line
Invalid line
Shortest valid line
Distance
Drug
Vascular
Mean distance =
(d1 + d2 + d3 + d4 + d5) / 5
b
FITC-CD31
Tmab-DL650
1 mm
c
d
Slice 20
Vascular (drug -)
Vascular (drug +)
Distance (95th percentile)
Distance (top 5 %)
f
Box plot of distance
Distance (μm)
Top 5 %
95th percentile
g
HIstogram of distance
Semi-log plot
Number of points
e
Distribution of mean distance
Mean distance (μm)
Slice number

Supplementary Fig. 19. Quantitative analysis of drug distribution in a tumor mass.

a. (Left) Method for identifying the nearest point on the vessel contour from the drug distribution contour The nearest-neighbor search was confined to the same drug distribution area belonged to the designated vessel. The lines traversing a non-drug area were considered invalid. (Right) Calculation of the mean drug distribution distance from the multiple valid line distances. If the designated point on a vessel contour was selected as the nearest-neighbor point from multiple points on the drug distribution contour, the final distance was defined as their mean value.

b. The whole *y*-*z* image of the CDX tumor mass (MIP viewed from the *x*-axis). The dashed lines depict representative slice positions shown in **c** and **d** among a total of 68 MIP slices from every 10 original *z*-stack slices. These MIP images, integrating *z*-stack information across ~100 µm thickness, were used for the following quantitative analysis.

c. Binarized composite images of the representative five MIP slices at the indicated positions in **b**, showing the drug and vessel distributions inside the tumor mass.

d. The quantitative analysis result of the drug distribution at the central part of the tumor mass (MIP slice #20). (Upper left) The binarized composite image of the selected slice. (Upper right) Vascular associating (magenta) or non-associating (yellow) with the drug distribution. The image was processed with dilate function in ImageJ/FIJI to enhance the vascular structure for visualization. (Lower left and right panels) The quantitative results of valid distance lines (values within 95th percentile range (gray) and top 5% (cyan), respectively), which are overlaid on the image shown in the upper right. The 95th percentile lines and the top 5% lines are mutually exclusive.

e. A box plot showing the drug distribution distances across all 68 MIP slices, indicating the region-dependent differences in drug distributions. The red lines represent the mean value, and the points indicate the outliers.

f. A box plot of all the valid line distances. The points indicate the outliers.

g. A histogram of all the valid line distances. The top 5% values are represented by cyan bars. Inset: a semi-logarithmic plot of the same data.

Supplementary Table 1. System components list of descSPIM

	Part number	Notes	Quantity
**Excitation optics**	CP33/M	Metric Threaded Cage Plate	3
	SM1FCA	SM1 to FC/APC Fiber Bulkhead Adapter	1
	CXY1A	High Precision Translating Lens Mount for 1" Optics	1
	AC254-100-A	f = 100 mm, Ø1" Achromatic Doublet, ARC: 400 - 700 nm	1
	KCB1EC/M	Metric SM1 Right Angle Kinematic Mirror Mount	3
	BBE1-E02	1" Broadband Dielectric Elliptical Mirror, 400 - 750 nm	3
	SM1V05	Ø1" Adjustable Lens Tube, 0.31" Travel Range	2
	SM1L10	SM1 Lens Tube, 1.00" Thread Depth, One Retaining Ring Included	1
	CT1A/M	13 mm Travel Manual Translation Stage for 30 mm Cage Systems	1
	LJ1144RM-A	f = 500.0 mm, Ø1", N-BK7 Mounted Plano-Convex Round Cyl Lens, ARC 350-700	1
	ACY254-150-A	f = 150.0 mm, Ø1" Cylindrical Achromat, AR Coating: 350 - 700 nm	1
	PH150/M	Ø12.7 mm Post Holder, Spring-Loaded Hex-Locking Thumbscrew, L = 150 mm	3
	PH50/M	Ø12.7 mm Post Holder, Spring-Loaded Hex-Locking Thumbscrew, L = 50 mm	1
	TR50/M-JP	Ø12 mm Optical Post, SS, M4 Setscrew, M6 Tap, L = 50 mm	4
	ER4-P4	Cage Assembly Rod, 4" Long, Ø6 mm, 4 Pack	4
	ER2-P4	Cage Assembly Rod, 2" Long, Ø6 mm, 4 Pack	2
	ER3-P4	Cage Assembly Rod, 3" Long, Ø6 mm, 4 Pack	1
	CPMA3	Snap-On 30 mm Cage Mounting Bracket, #8 (M4) Slot	4
**Sample holder**	XR25DR2	150 mm Long Rail for XR25	1
	XR25P/M	Metric Linear Translation Platform, 25mm	2
	Z825B	25 mm Motorized Actuator with Ø3/8" Barrel (0.5 m Cable)	1
	KDC101	K-Cube Brushed DC Servo Motor Controller	1
	KPS201	K-Cube Power Supply, 15V 2.4A, Japan	1
	PB1	Mounting Post Base 2.48 x 0.4 x 0.265	1
	P50/M	38mm Solid Post 50mm Length	1
	C1519/M	Metric Coarse Adjustable Height Platform	1
	MVS05/M	13mm Travel Manual Vertical Stage	1
	RLA300/M	Dovetail Optical Rail, 300 mm, Metric	2
	PR01/M	High-Precision Rotation Stage, M6 and M4 Taps	1
	ER2-P4	Cage Assembly Rod, 2" Long, Ø6 mm, 4 Pack	1
	ER4-P4	Cage Assembly Rod, 4" Long, Ø6 mm, 4 Pack	1
	SR1.5-P4	Compact Cage Assembly Rod, 1.5" Long, Ø4 mm, 4 Pack	1
	CP31/M	Blank 30 mm Cage Plate, 8.9 mm Thick, M4 x 0.7 Tap	1
	SP01	Blank 16 mm Cage Plate, 0.25" Thick, 4-40 and M3 Taps	1
	SP15/M	30 mm to 16 mm Cage Adapter Plate, M4 Tap	1
**Detection optics**	CS895MU	CMOS camera	1
	SM1A9	Adapter with External C-Mount Threads and Internal SM1 Threads	1
	SM1L10	SM1 Lens Tube, 1.00" Thread Depth, One Retaining Ring Included	1
	SM1L03	SM1 Lens Tube, 0.30" Thread Depth, One Retaining Ring Included	1
	SM1A2	Adapter with External SM1 Threads and Internal SM2 Threads	2
	TTL100-A	Tube Lens, f = 100 mm, ARC: 350 - 700 nm, External SM2 Threads	1
	SM1L15	SM1 Lens Tube, 1.50" Thread Depth, One Retaining Ring Included	1
	SM1QP	Fast-Change, SM1 Lens Tube Filter Holder for Filters 3.5 - 6.3 mm Thick	1
	SM1QT	Extra Filter Carriage for Filters 3.5 - 6.3 mm Thick	1
	FBH520-40	Premium Bandpass Filter, Ø25 mm, CWL = 520 nm, FWHM = 40 nm	1
	FBH550-40	Premium Bandpass Filter, Ø25 mm, CWL = 550 nm, FWHM = 40 nm	1
	FBH600-40	Premium Bandpass Filter, Ø25 mm, CWL = 600 nm, FWHM = 40 nm	1
	FBH700-40	Premium Bandpass Filter, Ø25 mm, CWL = 700 nm, FWHM = 40 nm	1
	FBLH0550	Ø25.0 mm Longpass Filter, Cut-On Wavelength: 550 nm	1
	SM1M05	SM1 Lens Tube Without External Threads, 1/2" Long, Two Retaining Rings Included	1
	SM1A12	Adapter with External SM1 Threads and Internal M25 x 0.75 Threads	1
	TL2X-SAP	2X obj lens (NA 0.1, w.d. 56.3 mm)	1
	SM1TC	Clamp for SM1 Lens Tubes and C-Mount Extension Tubes	2
	PH30/M	Ø12.7 mm Post Holder, Spring-Loaded Hex-Locking Thumbscrew, L=30 mm	2
	TR30/M-JP	Ø12 mm Optical Post, SS, M4 Setscrew, M6 Tap, L = 30 mm	2
	XR25P/M	25 mm Travel Linear Translation Stage, Side-Mounted Micrometer, M6 x 1.0 Taps	1
	Z825B	25 mm Motorized Actuator with Ø3/8" Barrel (0.5 m Cable)	1
	KDC101	K-Cube Brushed DC Servo Motor Controller (Power Supply Not Included)	1
	KPS201	K-Cube Power Supply, 15V 2.4A, Japan	1
**Others**	MB3045/M	Aluminum Breadboard, 300 mm x 450 mm x 12.7 mm, M6 Taps	1
	TPSM1/M	Magnetic Laser Safety Screen, 200 mm x 75 mm, Metric Engraving	1
	CPA2	30 mm Cage Alignment Plate with Ø5 mm Hole	1
	TC3/M	15-Piece Balldriver and Hex Key Kit with Stand, Metric	1
	SH6MS10	M6 x 1.0 Stainless Steel Cap Screw, 10 mm Long, 25 Pack	2
	SH4MS06	M4 x 0.7 Stainless Steel Cap Screw, 6 mm Long, 50 Pack	1
	SS6MS10	M6 x 1.0 Stainless Steel Setscrew, 10 mm Long, 25 Pack	1
	N6MS010	M6 x 1.0 Stainless Steel Nut, 50 Pack	1
**Besides Thorlabs**	-	Single-mode fiber (FC/APC) coupled laser ligh source (Cobolt skyra, 06 series or Coherent OBIS etc.)	1
	-	4-side cleared BK7 cuvette (Opt. Length 10 mm or 20 mm; GL Science etc)	1
	-	4-side cleared disposable cuvette (Opt. Length 10 mm; Thermo Fisher Scientific, Bio-Rad etc)	1

Supplementary References.

1. Stelzer, E. H. K. *et al.* Light sheet fluorescence microscopy. *Nat. Rev. Method. Prim.* **1**, 1–25 (2021).
2. Hobson, C. M. *et al.* Practical considerations for quantitative light sheet fluorescence microscopy. *Nat. Methods* **19**, 1538–1549 (2022).
3. Gao, L. Extend the field of view of selective plan illumination microscopy by tiling the excitation light sheet. *Opt. Express* **23**, 6102–6111 (2015).
4. Susaki, E. A. *et al.* Versatile whole-organ/body staining and imaging based on electrolyte-gel properties of biological tissues. *Nat. Commun.* **11**, 1982 (2020).
