# Supplementary material for: descSPIM: an affordable and easy-to-build light-sheet microscope optimized for tissue clearing techniques
Source: Nat Commun. 2024 Jun 12;15:4941. doi: 10.1038/s41467-024-49131-1 (PMC11169475; doi:10.1038/s41467-024-49131-1)
Supplement: Supplementary file 2 — Reporting Summary [file 41467_2024_49131_MOESM2_ESM.pdf]

nature portfolio

Corresponding author(s): Etsuo A Susaki

Last updated by author(s): May 10, 2024

Reporting Summary

Nature Portfolio wishes to improve the reproducibility of the work that we publish. This form provides structure for consistency and transparency in reporting. For further information on Nature Portfolio policies, see our Editorial Policies and the Editorial Policy Checklist.

Statistics

For all statistical analyses, confirm that the following items are present in the figure legend, table legend, main text, or Methods section.

n/a	Confirmed	
☐	☒	The exact sample size (*n*) for each experimental group/condition, given as a discrete number and unit of measurement
☐	☒	A statement on whether measurements were taken from distinct samples or whether the same sample was measured repeatedly
☒	☐	The statistical test(s) used AND whether they are one- or two-sided<br>*Only common tests should be described solely by name; describe more complex techniques in the Methods section.*
☒	☐	A description of all covariates tested
☒	☐	A description of any assumptions or corrections, such as tests of normality and adjustment for multiple comparisons
☐	☒	A full description of the statistical parameters including central tendency (e.g. means) or other basic estimates (e.g. regression coefficient) AND variation (e.g. standard deviation) or associated estimates of uncertainty (e.g. confidence intervals)
☒	☐	For null hypothesis testing, the test statistic (e.g. *F*, *t*, *r*) with confidence intervals, effect sizes, degrees of freedom and *P* value noted<br>*Give P values as exact values whenever suitable.*
☒	☐	For Bayesian analysis, information on the choice of priors and Markov chain Monte Carlo settings
☒	☐	For hierarchical and complex designs, identification of the appropriate level for tests and full reporting of outcomes
☒	☐	Estimates of effect sizes (e.g. Cohen's *d*, Pearson's *r*), indicating how they were calculated

Our web collection on statistics for biologists contains articles on many of the points above.

Software and code

Policy information about availability of computer code

Data collection	KInesis, ThorCam, ImageJ/fiji, µManager, Python, NIS-Elements Ar (5.02.03) for descSPIM images acquisition.<br>LabVIEW 2016 (National Instruments) for controlling another custom build light sheet microscope (Gemini).
Data analysis	Linux Ubuntu 20.04, Python 3-9-**, ANTs, ImageJ/fiji

For manuscripts utilizing custom algorithms or software that are central to the research but not yet described in published literature, software must be made available to editors and reviewers. We strongly encourage code deposition in a community repository (e.g. GitHub). See the Nature Portfolio guidelines for submitting code & software for further information.

Data

Policy information about availability of data

All manuscripts must include a data availability statement. This statement should provide the following information, where applicable:

- Accession codes, unique identifiers, or web links for publicly available datasets
- A description of any restrictions on data availability
- For clinical datasets or third party data, please ensure that the statement adheres to our policy

The datasets used in the current study are available from the corresponding authors on reasonable request because the raw data files collected by light-sheet microscope are too large to upload.

Research involving human participants, their data, or biological material

Policy information about studies with human participants or human data. See also policy information about sex, gender (identity/presentation), and sexual orientation and race, ethnicity and racism.

Reporting on sex and gender	Current study is not relevant.
Reporting on race, ethnicity, or other socially relevant groupings	Current study is not relevant.
Population characteristics	Current study is not relevant.
Recruitment	Current study is not relevant.
Ethics oversight	Current study is not relevant.

Note that full information on the approval of the study protocol must also be provided in the manuscript.

Field-specific reporting

Please select the one below that is the best fit for your research. If you are not sure, read the appropriate sections before making your selection.

☒ Life sciences ☐ Behavioural & social sciences ☐ Ecological, evolutionary & environmental sciences

For a reference copy of the document with all sections, see nature.com/documents/nr-reporting-summary-flat.pdf

Life sciences study design

All studies must disclose on these points even when the disclosure is negative.

Sample size	Sample size was n=5 for the mouse brain hemisphere imaging experiments (Fig. 2a), n=2 for the mouse brain coronal slice imaging experiments (Fig. 2b), the whole mouse brain experiments (Fig. 3, Supplementary Fig 17), the 3-color CDX imaging experiments (Fig. 4a) and the fluo-HE CDX imaging experiments (Fig. 4b), resulting in near identical results. In addition, sample size was n=1 for the rat-brain coronal slice imaging experiment (Fig. 5b), the mouse brain hemisphere imaging experiment (Fig. 5c), the mouse lung imaging experiment (Fig. 5d), the mouse stomach imaging experiment (Fig. 5e), the mouse intestine imaging experiment (Fig. 5f), the zebrafish imaging experiment (Fig. 5g), the E7.5 decidua imaging experiment (Fig. 5h) and the E12.0 whole mouse embryo tadpole imaging experiment (Fig. 5i). Sample sizes were chosen to give examples of microscope application, rather than do any specific biological claims.
Data exclusions	No data were excluded in the analyses.
Replication	The evaluation of PSF with fluorescent beads, quantum dots, and cleared PI-stained brains were repeated at least two independent samples. Most of the imaging experiments were repeated at least two independent measurement in the same or comparable condition with slight modification (e.g., laser light intensity, exposure time, and z-interval). All the results were reliably reproduced.
Randomization	The animals used for the experiments were randomly chosen from colonies. We do not claim any specific experimental findings, because our paper is focused on a new microscope design. Therefore, randomization is not relevant to our study.
Blinding	No blinding was done in this study because knowledge of experimental conditions during data collection was required . Quantitative Analyses was conducted using semi-automated workflow.

Reporting for specific materials, systems and methods

We require information from authors about some types of materials, experimental systems and methods used in many studies. Here, indicate whether each material, system or method listed is relevant to your study. If you are not sure if a list item applies to your research, read the appropriate section before selecting a response.

Materials & experimental systems

n/a	Involved in the study	
☐	☒	Antibodies
☐	☒	Eukaryotic cell lines
☒	☐	Palaeontology and archaeology
☐	☒	Animals and other organisms
☒	☐	Clinical data
☒	☐	Dual use research of concern
☒	☐	Plants

Methods

n/a	Involved in the study	
☒	☐	ChIP-seq
☒	☐	Flow cytometry
☒	☐	MRI-based neuroimaging

Antibodies

Antibodies used	FITC-labeled anti-murine CD31 antibody (BioLegend, CA, USA, #102506)<br>Trastuzumab (Herceptin®, Chugai Pharmaceutical)<br>Anti-tyrosine hydroxylase antibody (Merck, Darmstadt, Germany, MAB318)<br>Alexa Fluor 488-conjugated goat Fab for anti-mouse IgG1 Fc-specific (Jackson Immuno Research lab., 115-547-185)<br>Goat anti-chicken IgY Fab fragment Alexa 647 conjugate (Jackson, # 103-607-008)<br>Anti-β-galactosidase antibody (Abcam, #ab9361)<br>Goat anti-rabbit IgG Fab fragment Cy3 conjugate (Jackson, #111-167-008)<br>Anti-cleaved Caspase3 antibody (Cell Signaling Technology, #9661)
Validation	FITC-labeled anti-murine CD31 antibody validation has been done according to the vender's information on reacting species and applications. The vender's website is as below:<br>https://www.biolegend.com/ja-jp/products/fitc-anti-mouse-cd31-antibody-377?GroupID=BLG10531<br>Trastuzumab is commercially available as a antibody drug and has undergone strict quality checks by the pharmaceutical company. In using trastuzumab for imaging in this study, the binding of trastuzumab to the antigen was confirmed by staining positive and negative controls with a secondary antibody.<br>Anti-tyrosine hydroxylase antibody validation has been done according to the vender's information on reacting species and applications. The vender's website is as below:<br>https://www.sigmaaldrich.com/JP/ja/product/mm/mab318?utm_source=google&utm_medium=cpc&utm_campaign=19475204102&utm_content=143558715063&gclid=EAIaIQobChMIkbjrh8nshAMVwwd7Bx2LXQOTEAAYASAAEgJxhfD_BwE<br>Alexa Fluor 488-conjugated goat Fab for anti-mouse IgG1 Fc-specific validation has been done according to the vender's information on reacting species and applications. The vender's website is as below:<br>https://www.jacksonimmuno.com/catalog/products/115-547-185<br>Goat anti-chicken IgY Fab fragment Alexa 647 conjugate validation has been done according to the vender's information on reacting species and applications. The vender's website is as below:<br>https://www.jacksonimmuno.com/catalog/products/103-607-008<br>Anti-β-galactosidase antibody validation has been done according to the vender's information on reacting species and applications. The vender's website is as below:<br>https://www.abcam.com/en-mt/products/primary-antibodies/beta-galactosidase-antibody-ab9361<br>Goat anti-rabbit IgG Fab fragment Cy3 conjugate validation has been done according to the vender's information on reacting species and applications. The vender's website is as below:<br>https://www.jacksonimmuno.com/catalog/products/111-167-008<br>Anti-cleaved Caspase3 antibody validation has been done according to the vender's information on reacting species and applications. The vender's website is as below:<br>https://www.cellsignal.jp/products/primary-antibodies/cleaved-caspase-3-asp175-antibody/9661

Eukaryotic cell lines

Policy information about cell lines and Sex and Gender in Research

Cell line source(s)	BT-474 human breast cancer cells (American Type Culture Collection, Manassas, USA, HTB-20)<br>G-292 clone A141B1 (JCRB cell bank #IFO50107)
Authentication	Cell lines are routinely verified by short tandem repeat analysis to be derived from the correct donor and are not contaminated.
Mycoplasma contamination	No mycoplasma contamination
Commonly misidentified lines<br>(See ICLAC register)	The cell line used in this study is not included in commonly misidentified lines.

Animals and other research organisms

Policy information about studies involving animals; ARRIVE guidelines recommended for reporting animal research, and Sex and Gender in Research

Laboratory animals	8- and 14-week-old male C57BL/6N mice (Japan SLC, Inc.)<br>9-week-old female Thy1-YFP-H Tg mice (B6.Cg-Tg(Thy1-YFP)HJrs/J, The Jackson Laboratory, Identifier: 003782)<br>35-week-old female Thy1-GFP-M Tg mice (STOCK Tg(Thy1-EGFP)MJrs/J, The Jackson Laboratory, Identifier: 007788)<br>5 to 6-week-old female SCID-beige mice (CB17.Cg-PrkdcscidLystbg-J/ CrlCrlj, Charles River Laboratories)<br>8-week-old male Sprague-Dawley rat (KBT Oriental) (Saga, Japan)<br>9-month-old female Camk2-tTA Tg mouse (B6.Cg-Tg(Camk2a-tTA)1Mmay/DboJ, The Jackson Laboratory, Identifier: 007004)<br>8-week-old male Chat-Cre mice (B6;129S6-Chattm2(cre)Lowl/J, The Jackson Laboratory, Identifier: 006410)<br>8-week-old male R26-LSL-TdTomato mice (B6.Cg-Gt(ROSA)26Sortm14(CAG-tdTomato)Hze/J, The Jackson Laboratory, Identifier: 007914)<br>8-week-old male Dclk1-Zsgreen mice (Ref: Middelhof et al., Nat Commun. 2020. doi: 10.1038/s41467-019-13850-7.)<br>a 10 week-old male mouse of in-house breeding originated from C57BL/6J (The Jackson Laboratory Japan, Inc.)

	Tg(fli1a:myr-EGFP)ncv2Tg zebrafish line (Ref: Fukuhara et al., Dev. Biol. 2014. doi: 10.1016/j.ydbio.2014.06.015)<br>10-week-old female pregnant ICR mice (Slc:ICR, Japan SLC, Inc.)<br>12-day post coitum mouse embryos of RARE-lacZ Transgenic (Tg) mouse (provided by RIKEN BioResource Research Center, #RBRC06571)
Wild animals	No wild animal was used.
Reporting on sex	Sex of all mice used in this study were randomly selected.
Field-collected samples	No field-collected samples were used.
Ethics oversight	All experimental procedures and housing conditions of the animals were approved by the Animal Care and Use Committees of Juntendo University (1569-2022279 and 1372-2022211), National Cancer Center Research Institute (T21-012), Brain Research Institute, Niigata University (SA01266), National Institute for Physiological Sciences (22A044), Kagoshima University (VM21058), the National Institutes for Quantum Science and Technology (23-1032; R5-10-1), the Japanese Foundation for Cancer Research (10-01-22), the University of Tokyo (A2023M095), Central Research Institute of Electric Power Industry (2305), the Nippon Medical School (2022-020), Kyushu University (A22-029-4), and Kyoto Prefectural University of Medicine (M2023-178). All of the animals were cared for and treated humanely in accordance with the Institutional Guidelines and with the recommendations of the United States National Institutes of Health for experiments using animals.

Note that full information on the approval of the study protocol must also be provided in the manuscript.

Plants

Seed stocks	*Report on the source of all seed stocks or other plant material used. If applicable, state the seed stock centre and catalogue number. If plant specimens were collected from the field, describe the collection location, date and sampling procedures.*
Novel plant genotypes	*Describe the methods by which all novel plant genotypes were produced. This includes those generated by transgenic approaches, gene editing, chemical/radiation-based mutagenesis and hybridization. For transgenic lines, describe the transformation method, the number of independent lines analyzed and the generation upon which experiments were performed. For gene-edited lines, describe the editor used, the endogenous sequence targeted for editing, the targeting guide RNA sequence (if applicable) and how the editor was applied.*
Authentication	*Describe any authentication procedures for each seed stock used or novel genotype generated. Describe any experiments used to assess the effect of a mutation and, where applicable, how potential secondary effects (e.g. second site T-DNA insertions, mosiacism, off-target gene editing) were examined.*
