# Supplementary material for: descSPIM: an affordable and easy-to-build light-sheet microscope optimized for tissue clearing techniques
Source: Nat Commun. 2024 Jun 12;15:4941. doi: 10.1038/s41467-024-49131-1 (PMC11169475; doi:10.1038/s41467-024-49131-1)
Supplement: Supplementary file 4 — Description of Additional Supplementary Files [file 41467_2024_49131_MOESM4_ESM.pdf]

Description of Additional Supplementary Files for

# descSPIM: an affordable and easy-to-build light-sheet microscope optimized for tissue clearing techniques

**Supplementary Movie 1.**

3D reconstructed image of PI-stained mouse brain hemisphere, shown in **Fig. 2a**.

**Supplementary Movie 2.**

3D reconstructed image of PI-stained 2-mm thick mouse brain section, shown in **Fig. 2c**.

**Supplementary Movie 3.**

Two-color, 3D reconstructed image of PI-stained whole-brain image of *Thy1*-YFP-H Tg mouse, shown in **Fig. 3a**.

**Supplementary Movie 4.**

Three-color, 3D reconstructed image of Trastuzumab-administered CDX sample, shown in **Fig. 4a.**

**Supplementary Movie 5.**

The quantitative analysis result of the drug distribution at the central part of the tumor mass, shown in **Supplementary Fig. 19d**.
Max intensity projection images of approximately 100 µm thickness of Trastuzumab-administered CDX sample. FITC-labeled CD31 (yellow) and DyLight 650™-labeled Trastuzumab (magenta) are shown of the raw images (**a**), and of the processed binarized images (**b**). **c**. Vascular associating (magenta) or non-associating (yellow) with the drug distribution. **d**. The valid distance lines (values within 95th percentile range (gray) and top 5% (cyan), respectively), which are overlaid on the image shown in **c**.

**Supplementary Movie 6.**

Cross-sectional false-colored hematoxylin and eosin images of CDX sample, shown in **Fig. 4b**.
